# Supplementary material for: Impact of Feed Composition on Rumen Microbial Dynamics and Phenotypic Traits in Beef Cattle
Source: Microorganisms. 2025 Jan 31;13(2):310. doi: 10.3390/microorganisms13020310 (PMC11857910; doi:10.3390/microorganisms13020310)
Supplement: Supplementary file 1 [file microorganisms-13-00310-s001.zip › microorganisms-3391585-supplementary.pdf]

Supporting information

Table S1. Experimental design included four pens of fifty-nine bulls fed either forage or grain-based diets over two periods

Pens[1]	Period 1 (0 - 80 Days)	Period 2 (100-180 Days)
1 (n= 15 bulls)	Forage	Forage
2 (n= 15 bulls)	Forage	Grain
3 (n= 14 bulls)	Grain	Forage
4 (n= 15 bulls)	Grain	Grain

[1]Rumen fluid samples were collected on days 0 and 80 in Period 1 and on days 100 and 180 in Period 2, with a 20-day adaptation phase between periods (See Materials and Methods for details).

Table S2. Ingredient and chemical composition of the forage and grain diets

	Diets	
Item	Forage	Grain
**Ingredient composition, %, as-fed basis**		
Alfalfa Hay	17.9	-
Corn Silage	81.6	39
Alfalfa Silage	-	33.1
Corn Grain	-	27.7
Limestone	0.3	-
Mineral	0.1	0.1
Salt	0.1	0.1
**Chemical composition, DM basis**		
Dry Matter, %	50.7	52.7
Acid Detergent Fiber, %	21.78	15.63
Neutral Detergent Fiber, %	40.89	27.16
Total Digestible Nutrients, %	73.51	79.87
Starch, %	21.6	31.3
Metabolizable Energy, MJ kg^{-1}	11.10	12.06
Crude Protein, %	13.1	12.3
Calcium, %	0.89	0.77
Phosphorus, %	0.45	0.42
Magnesium, %	0.42	0.34
Potassium, %	2.16	1.65
